# Isolated effects of patellar resurfacing in total knee arthroplasty and their relation to native patellar geometry

**DOI:** 10.1038/s41598-022-16810-2

**Published:** 2022-07-28

**Authors:** Adrian Sauer, Christoph Thorwaechter, Ingrid Dupraz, Allan Maas, Arnd Steinbrueck, Thomas M. Grupp, Matthias Woiczinski

**Affiliations:** 1 Aesculap AG, Research and Development, Am Aesculap-Platz, 78532 Tuttlingen, Germany; 2 Ludwig Maximilians University Munich, Department of Orthopaedic and Trauma Surgery, Musculoskeletal University Center Munich (MUM), University Hospital, Munich, Germany; 3 Orthopaedic Surgical Competence Center Augsburg (OCKA), Augsburg, Germany

**Keywords:** Musculoskeletal system, Osteoarthritis, Orthopaedics

## Abstract

The isolated effects of patellar resurfacing on patellar kinematics are rarely investigated. Nonetheless, knowing more about these effects could help to enhance present understanding of the emergence of kinematic improvements or deteriorations associated with patellar resurfacing. The aim of this study was to isolate the effects of patellar resurfacing from a multi-stage in vitro study, where kinematics after total knee arthroplasty before and after patellar resurfacing were recorded. Additionally, the influence of the native patellar geometry on these effects was analysed. Eight fresh frozen specimens were tested successively with different implant configurations on an already established weight bearing knee rig. The patello-femoral kinematics were thereby measured using an ultrasonic measurement system and its relation to the native patellar geometries was analysed. After patellar resurfacing, the specimen showed a significantly medialized patellar shift. This medialization of the patellar tracking was significantly correlated to the lateral facet angle of the native patella. The patellar shift after patellar resurfacing is highly influenced by the position of the patellar button and the native lateral patellar facet angle. As a result, the ideal medio-lateral position of the patellar component is affected by the geometry of the native patella.

## Introduction

In total knee arthroplasty (TKA), the question of whether or not to resurface the patella remains controversial^1,2^. There is evidence to indicate that patellar resurfacing can lead to both improved clinical outcomes and a reduced probability of revision surgery^1,3,4^. To identify and prove the effects of patellar resurfacing, functional analyses are shown to be more suitable than the established knee scores^5^.

Detecting the isolated effects of patellar resurfacing on kinematics after TKA is a difficult task, mainly due to the high variation between subject kinematics. In order to address this limitation, a study aiming to characterize these effects must evaluate post-operative kinematics for a large enough cohort of patients, or, alternatively, assess knee kinematics after TKA both before and after patellar resurfacing. As a result, the studies that take on this objective are few and far between. Nevertheless, it is known that while patellar resurfacing has only minor effects on tibio-femoral kinematics^6,7^ or gait patterns^8,9^, it has been shown to influence patello-femoral kinematics^10^ and tibio-femoral range of motion^11^. Additionally, it is possible to improve abnormal pre-operative patello-femoral motion patterns by navigated patellar resurfacing^12^.

There are recommendations to medialize the patellar component on the patellar cut to restore the position of the patellar ridge^13^, but since this medialization can cause an increase of lateral patellar tilt for some patients^14^, the effect of the native patellar geometry on the optimal position of the patellar component needs to be further analysed.

The aim of this study was firstly to quantify the isolated effect of patellar resurfacing on patellar kinematics in an in vitro test setup for two different posterior stabilized implant variants. The hypotheses were that patellar kinematics are significantly influenced by patellar resurfacing but not by changing the tibiofemoral implant variants. Secondly the found effects were analysed and related to the native patellar geometries to enable a more nuanced view on the ideal placement of the patellar component in TKA.

## Materials and methods

### Specimens and implantation

For this study, eight fresh frozen specimens, (5 male, 3 female, age 52 ± 16 years, 7 left and 1 right) with less than 10° of varus or valgus and without severe bone deformities, were used. The implantation and testing process was the same as described in earlier studies^15–20^. The tibia and femur were cut 22 cm and 20 cm from the epicondylar line, respectively, and were embedded into pots with epoxy resin (RenCast FC 52/53 Isocyanate & FC 53 Polyol, Huntsman Advanced Materials GmbH, Texas, USA) The fibula head was fixed in the proximal tibia with a cortical screw. The vastus medialis, vastus lateralis, semitendinosus, rectus femoris and biceps femoris were attached to metallic finger traps (Bühler-Instrumente Medizintechnik GmbH, Tuttlingen, Germany) to apply muscle forces in the knee rig.

The posterior stabilized implants (Vega PS System, Aesculap AG, Tuttlingen, Germany) were placed in tibia-first technique with an intramedullary alignment. The femoral medio-lateral position was centred to the femoral bone, while its rotation was aligned to the anatomical trans-epicondylar axis. The used femoral implant has the same radius in the cavity of the trochlea as the dome-shaped patellar button. Therefore, full congruency is reached in the artificial patello-femoral joint. To increase the resistance against lateral subluxation of the patella, the lateral flange is slightly more pronounced than the medial one.

To implant the patellar button in a second surgery step after kinematic testing of the native patella, the thickness of the patellar button was resected and the medio-lateral position of the patellar implant was centred on the surface of the bone cut. No medialization of the patellar button was performed.

With every specimen several trials were executed. Before implantation the native knee was tested. After the implantation of the tibiofemoral implants, trials with a PS and a PS+ inlay were executed with the native and resurfaced patella, respectively.

### Knee rig

A well-established weight bearing knee rig^15–20^ which performs a squat from 30° to 130° moving 3°/s, was used for testing. To measure the flexion angle, two angular sensors (8820, Burster, Gernsbach, Germany) placed at the hip and ankle joint were used. For each of the three bone segments of the knee joint, the movements of four bony landmarks were recorded with an ultrasonic measurement system (Zebris CMS20, Isny, Germany). These landmarks allow the mapping of the anatomic reference frames to the femur, tibia and patella bones.

The resolution of the sensors is 0.1° and 0.1 mm. The reliability was measured proviously with seven specimen, three cycles each, where the mean standard deviation was 0.2 mm for patellar shift, 0.5° for patellar flexion and 0.2° for patellar spin and tilt, respectively.

**Figure 1:**
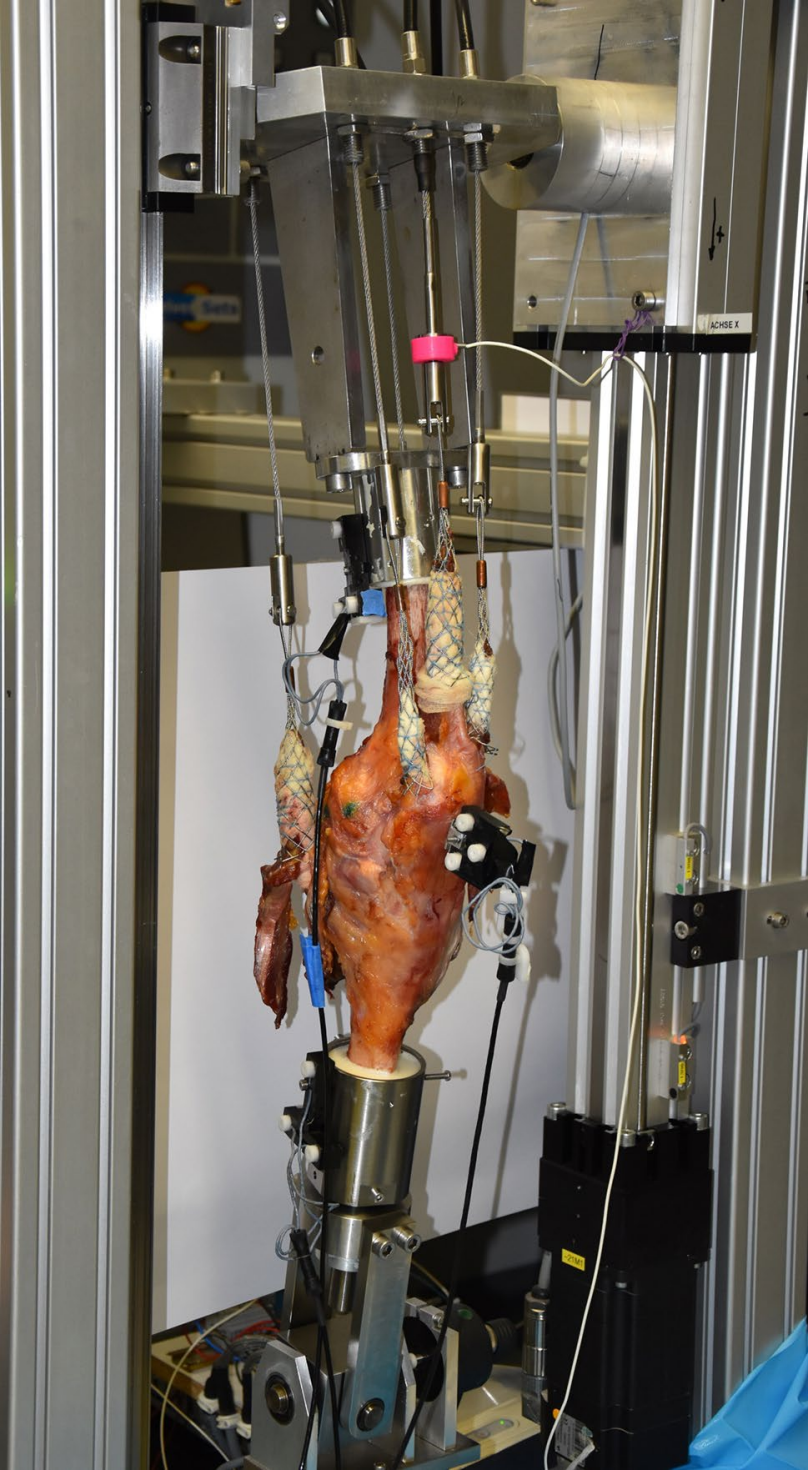
One of the specimens mounted on the knee rig.

The rig is shown in Fig. 1 and applies passive forces of 20 N to the vastus medialis, vastus lateralis, semitendinosus and the biceps femoris, while the force of the rectus femoris is actively controlled to reach 50 N of ground reaction force at the ankle joint (FN 7325–31, FGP Sensors & Instrumentation, Les Clayes Sous Bois, France). LabView was used for real time test control (Version 8.6, National Instruments, Austin, TX, USA).

### Data analysis

From the recorded trajectories of the landmarks, the patello-femoral kinematics were calculated for every trial using Matlab (MathWorks Inc., Natick, MA, USA). Patello-femoral kinematics are described using the three-cylinder-open-chain method^21,22^, where a positive patellar shift is given if the patella translates along the femoral flexion axis to the lateral side. Patellar flexion, tilt and spin are the rotations of the patella around the femoral flexion axis, the patellar long-axis and the floating axis between these two, respectively.

Since effects caused by the differences between the specimens would cover effects of the different implant configurations, only differences to the native situation, termed relative kinematics, are analysed within this study.

**Figure 2:**
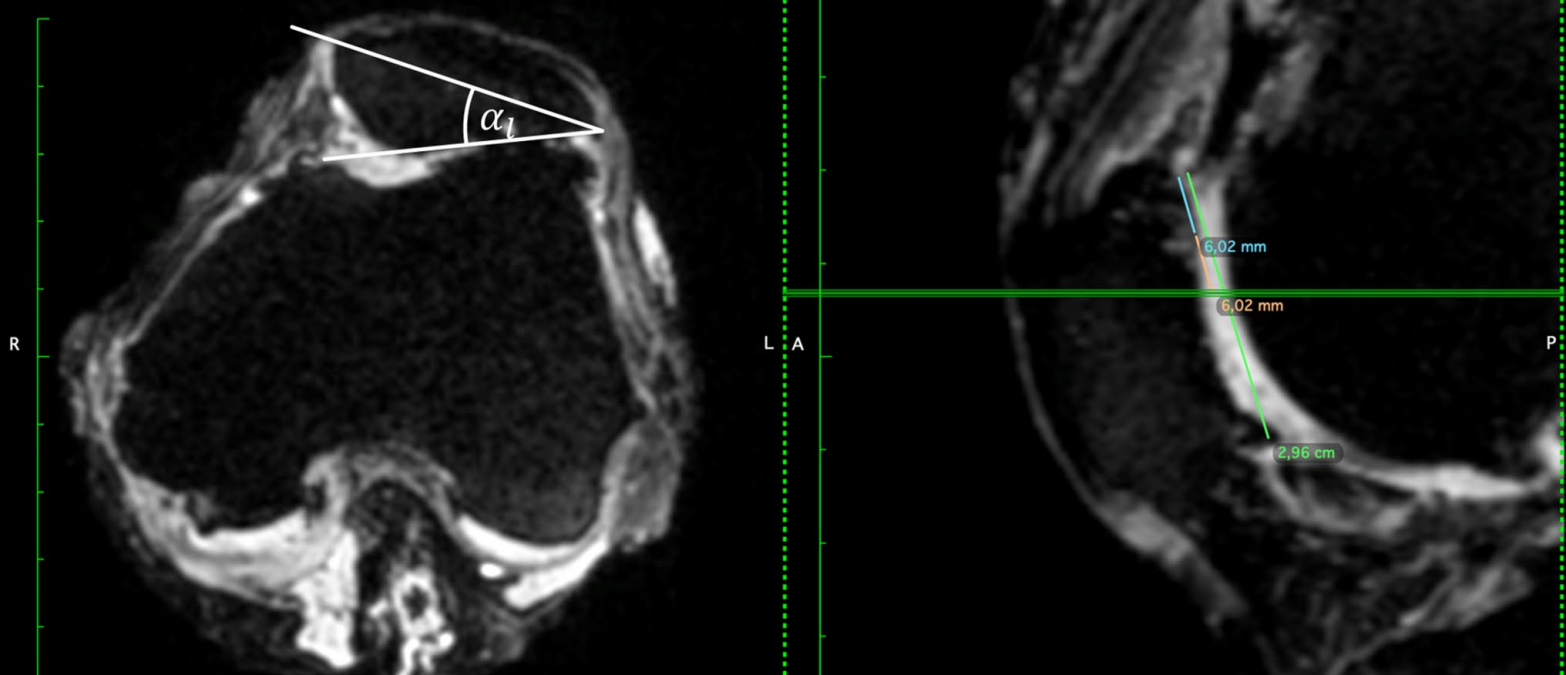
Visualization of the angles measured in the MRI data of the native knee joint. Left: patello-femoral joint in view from distal with measured lateral patellar facet angle. Right: sagittal view with the actual plane for the distal view (green). The angle drawn in the left image was measured in four parallel and equidistant planes of the patella.

For every specimen, an MRI-scan (Siemens Aera, Siemens AG, Munich, Germany) was executed. The native MRI was executed with frozen specimens to avoid an additional defrosting cycle. Therefore, it was not possible to control the knee flexion angle in an accurate manner for the MRI scans. All scans were executed between full extension and 30° of flexion. Four parallel planes, which were orthogonal to the sagittal plane and equidistantly distributed between the proximal and distal end of the patella, were defined within these scans. For these planes, the angle between the tangent of the lateral facet and the patellar plane was measured, as shown in Fig. 2. The average of all measurements for every specimen was used as the lateral patellar facet angle α_l_.

A linear regression model was calculated to analyse the relationship between the lateral patellar facet angle and the difference in patellar shift. An F-Test was executed to test if the slope of this regression function differs significantly from zero.

### Ethics statement

This study was approved by the ethics committee of the University of Munich, Germany (approval 58–16) and carried out in accordance with relevant guidelines and regulations. Informed consent for donation to scientific research had been signed before death by the donors or after death by their relatives.

## Results

**Figure 3:**
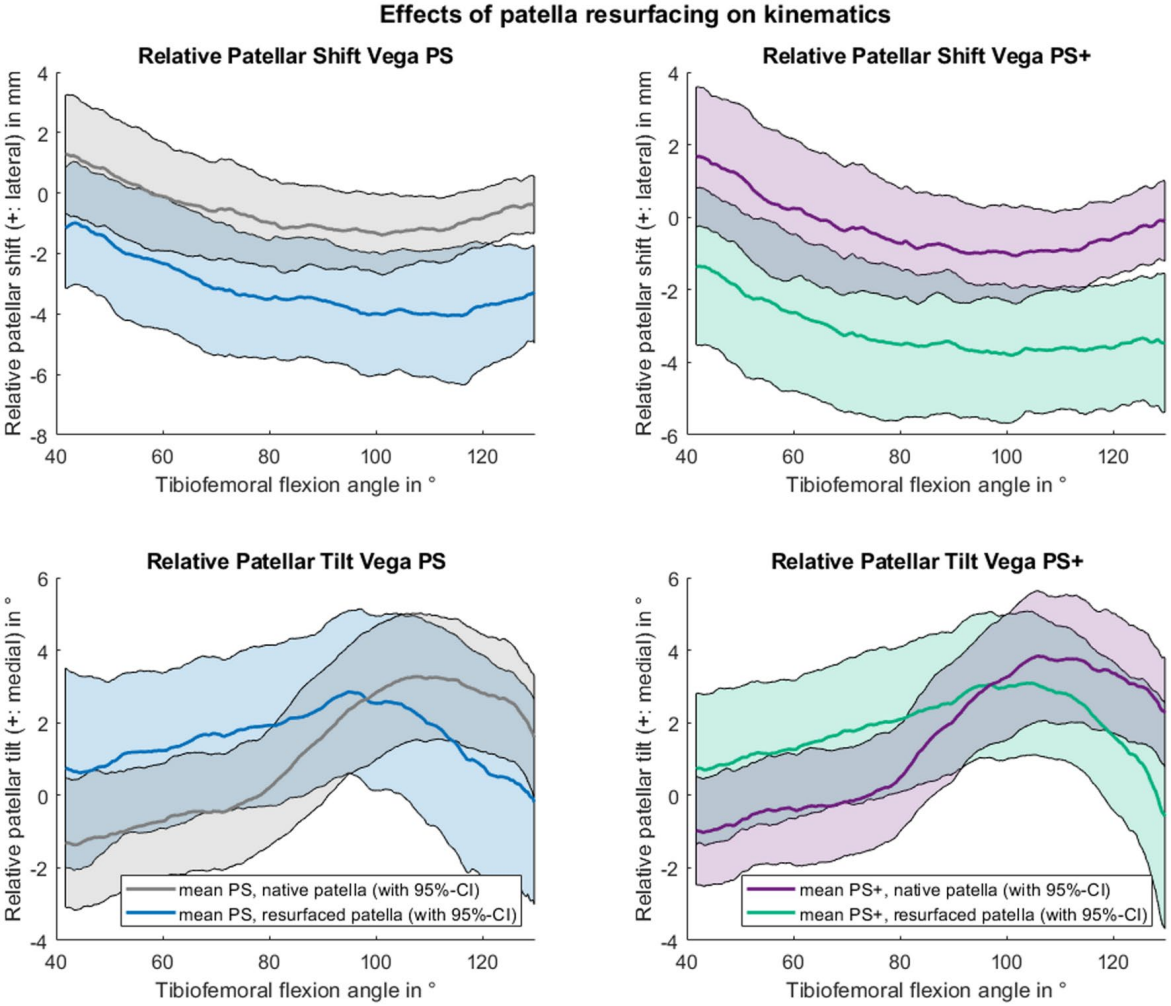
Differences to the native situation for patellar shift (top) and tilt (lower) with Vega PS (left) and PS+ (right) inserts for native (grey/purple) and resurfaced patella (blue/green).

For the patellar kinematics relative to the native knee, no significant differences between the Vega PS and PS+ inserts were found. The comparison between the implant configurations with resurfaced and native patella also showed no significant differences for patellar rotations (patellar flexion, tilt and spin). The average curve of the patellar shift for all specimens with resurfaced patella is outside of the 95%-confidence intervals (CI) for native patella and vice versa, as shown in Fig. 3. Therefore, the patellar shift after patellar resurfacing is significantly lower than before, indicating a more medial patellar tracking after resurfacing. The averaged medialization for the full load cycle after patellar resurfacing is 2.7 ± 1.2 mm. For these isolated effects of patellar resurfacing, no significant difference was found between male and female specimens.

**Figure 4:**
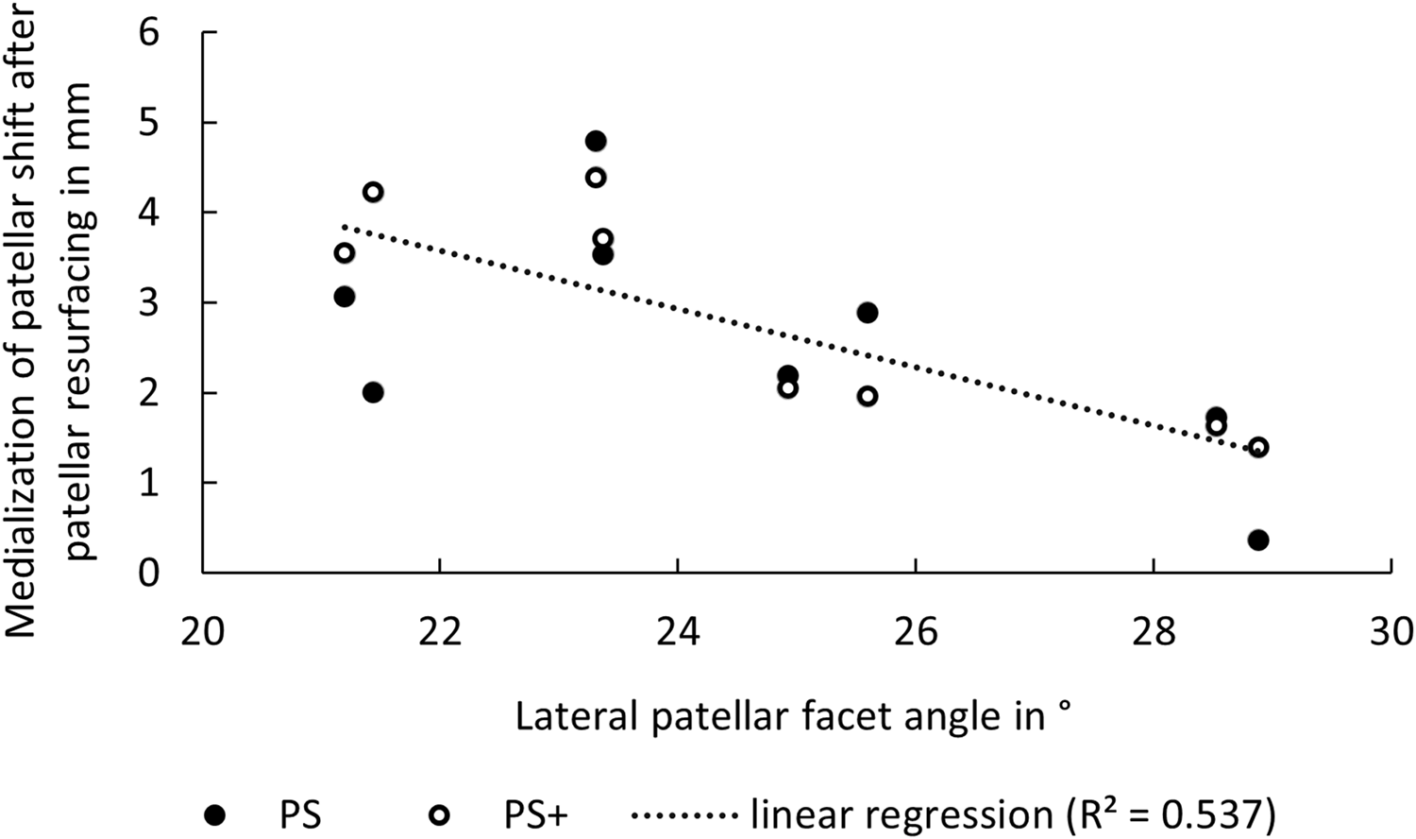
Dot plot showing the connection between the average difference of patellar shift before and after patellar resurfacing and the lateral patellar facet angle for PS and PS+. The linear regression model for all these values shows, that 53.7% of the variance in the patellar shift difference can be explained by the lateral facet angle.

In a linear regression model, the lateral facet angle explains 72.9% (PS+) and 39.4% (PS) of the variance of the averaged difference in the patellar shift before and after patellar resurfacing. If Vega PS and PS+ are combined in one dataset, 53.7% of the variance in the averaged patellar shift difference were explained by a (highly) significant (p = 0.001) linear regression function of the lateral facet angle. A higher lateral facet angle is related to a more lateral patella tracking after resurfacing and to a decreased difference in patellar shift before and after patellar resurfacing. The data and linear regression function are shown in Fig. 4.

## Discussion

The objective of this study was to analyse patello-femoral kinematics and how they are influenced by differing implant configurations, with a special focus given to the effects of patellar resurfacing. The posterior-stabilized Vega implants were tested with PS and PS+ inlays before and after patellar resurfacing, respectively. The difference in the PS and PS+ tibial inlays did not change patello-femoral kinematics in a significant way, either in the native stage nor in the patellar resurfaced stage. Placing the patellar button significantly affected patellar tracking, which was medialized with the resurfaced patella. Patello-femoral rotations were not significantly affected by patellar resurfacing.

Patellar resurfacing led to a significant difference in patellar shift. For a given femoral implant, a resurfaced patella will, on average, be medialized by 2.7 ± 1.2 mm when compared to the native patella. Ostermeier et al. came to similar results in their in vitro study, where the maximum medialization of the patella increased by 3 mm after resurfacing even though the patella was placed with 5 mm of medialization^10^.

**Figure 5:**
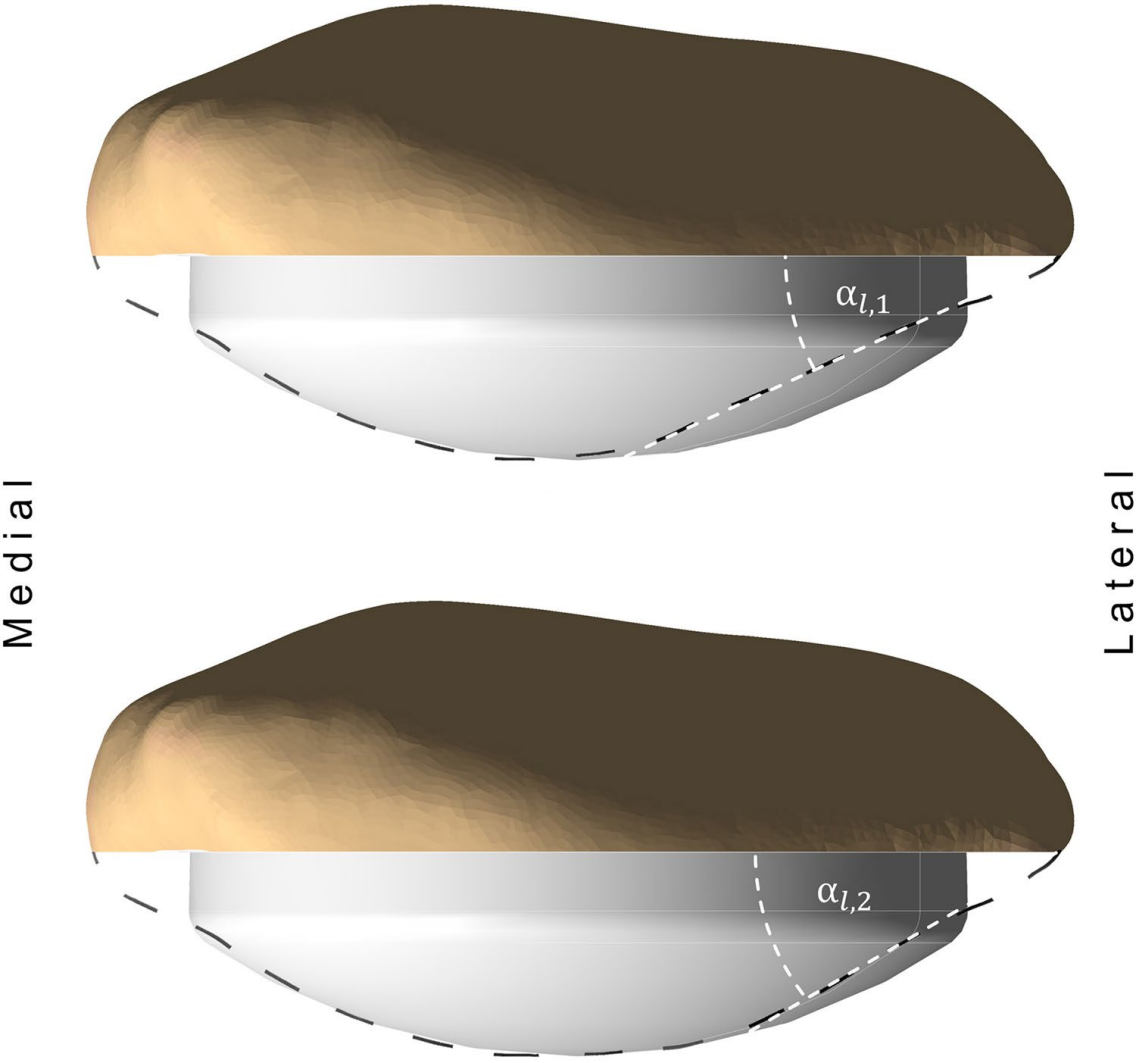
Lateral overstuffing for patellae with different native lateral facet angles with a centrally placed dome-shaped patellar component. For a small lateral facet angle α_l,1_, the lateral overstuffing increases (top) compared to a bigger native lateral facet angle α_l,2_ (lower).

The present study has demonstrated that the described difference in patellar shift can be associated with the lateral patellar facet angle, such that 53.7% of patellar shift variance can be explained by this specific parameter. This relationship can be caused by the underlying surgical protocol, where the patellar button position was centred on the bone cut surface. Using this method, a small lateral facet angle leads to an overstuffing of the patellar button on the native lateral facet, as is illustrated in Fig. 5. Therefore, the patella is forced to be medialized.

The present study has some limitations. Firstly, the deep knee bend was performed with a constant ground reaction force of 50 N and secondly, it must be mentioned that this is an in-vitro study with a small sample size. Furthermore, measuring the native lateral patellar facet angle can be very inaccurate for cases with advanced arthritis. This would prevent surgeons from applying the results of this study.

From our study, we can conclude that resurfacing the patella without medialization could lead to a change in patellar shift. For specimens with pronounced patellar facet asymmetry, where the medial facet is more steep than the lateral one, this effect seems to be particularly evident. To reduce changes in patellar shift, the patellar button can be placed with a tendency towards the side, where the native facet with the higher angle was located. Since the medial facet angle usually tends to be higher than the lateral one, a slight offset of the patellar button to the medial side, as has been often recommended^23,24^, shall prove to be a favourable choice in the majority of cases. It was reported earlier that medialization of the patellar component can reduce the patellofemoral contact force and lateral retinacular tension, as well as the frequency of its release^25^. This could be explained by the described lateral overstuffing of the patellar component and the resulting medialization of the patella tracking found in this study.

A surgeon’s first inclination to avoid lateral subluxation of the patella might be to favour a lateral placement of the patellar button, which would lead to overall medialization of the patella tracking. However, the results of this study show that, in fact, this medialization of the entire patella can just as successfully be achieved by opting for a central button positioning. This effect was especially notable in cases where the lateral patellar facet angle was small.

## Conclusions

Based on our results, it could be worth to consider the native patellar geometry to choose the medio-lateral position of the patellar implant, and thus minimize the deviation from patellar kinematics without patellar resurfacing. Further research needs to be done to analyse how to determine the medio-lateral implant position in everyday clinical workflows.

## Data Availability

The datasets generated and analysed during the current study are available from the corresponding author on reasonable request.
